# A novel approach for next‐generation sequencing of circulating tumor cells

**DOI:** 10.1002/mgg3.210

**Published:** 2016-02-28

**Authors:** Stephanie S. Yee, David B. Lieberman, Tatiana Blanchard, JulieAnn Rader, Jianhua Zhao, Andrea B. Troxel, Daniel DeSloover, Alan J. Fox, Robert D. Daber, Bijal Kakrecha, Shrey Sukhadia, George K. Belka, Angela M. DeMichele, Lewis A. Chodosh, Jennifer J. D. Morrissette, Erica L. Carpenter

**Affiliations:** ^1^ Department of MedicineUniversity of Pennsylvania Perelman School of MedicinePhiladelphiaPennsylvania; ^2^ Department of Pathology and Laboratory MedicineHospital of the University of PennsylvaniaPhiladelphiaPennsylvania; ^3^ Department of Cancer BiologyUniversity of Pennsylvania Perelman School of MedicinePhiladelphiaPennsylvania; ^4^ Abramson Family Cancer Research InstituteUniversity of Pennsylvania Perelman School of MedicinePhiladelphiaPennsylvania; ^5^ Division of OncologyCenter for Childhood Cancer ResearchChildren's Hospital of PhiladelphiaPhiladelphiaPennsylvania; ^6^ Center for Clinical Epidemiology and BiostatisticsUniversity of PennsylvaniaPhiladelphiaPennsylvania; ^7^ Abramson Cancer CenterUniversity of Pennsylvania Perelman School of MedicinePhiladelphiaPennsylvania

**Keywords:** Breast cancer, circulating tumor cell, liquid biopsy, next‐generation sequencing, personalized medicine, whole genome amplification

## Abstract

**Background:**

Next‐generation sequencing (NGS) of surgically resected solid tumor samples has become integral to personalized medicine approaches for cancer treatment and monitoring. Liquid biopsies, or the enrichment and characterization of circulating tumor cells (CTCs) from blood, can provide noninvasive detection of evolving tumor mutations to improve cancer patient care. However, the application of solid tumor NGS approaches to circulating tumor samples has been hampered by the low‐input DNA available from rare CTCs. Moreover, whole genome amplification (WGA) approaches used to generate sufficient input DNA are often incompatible with blood collection tube preservatives used to facilitate clinical sample batching.

**Methods:**

To address this, we have developed a novel approach combining tumor cell isolation from preserved blood with Repli‐G WGA and Illumina TruSeq Amplicon Cancer Panel‐based NGS. We purified cell pools ranging from 10 to 1000 cells from three different cell lines, and quantitatively demonstrate comparable quality of DNA extracted from preserved versus unpreserved samples.

**Results:**

Preservation and WGA were compatible with the generation of high‐quality libraries. Known point mutations and gene amplification were detected for libraries that had been prepared from amplified DNA from preserved blood.

**Conclusion:**

These spiking experiments provide proof of concept of a clinically applicable workflow for real‐time monitoring of patient tumor using noninvasive liquid biopsies.

## Introduction

Comprehensive molecular characterization of solid tumor samples is a common practice for detection of therapeutically targetable genetic lesions and guidance of clinical decision making (Vignot et al. 2013; Shaw et al. 2014; Wheler et al. 2014; Wilson et al. 2014; Azzato et al. 2015). Advances in the care of breast cancer patients have included the use of pharmaceutical agents to target specific genetic lesions such as the use of pertuzumab and trastuzumab to treat *HER2*‐amplified tumors (Valero et al. 2011; Swain et al. 2015). Promising drugs to treat tumors harboring *PIK3CA* mutations and/or activation are being evaluated in early phase trials (Mayer et al. 2014; Saura et al. 2014). Molecular tumor profiling can also facilitate the detection of mechanisms of resistance to therapy, such as the emergence of *ESR1* mutations in response to estrogen receptor‐targeted therapy (Robinson et al. 2013; Toy et al. 2013). Finally, comprehensive genetic analysis can be used to assess tumor heterogeneity (Gerlinger et al. 2012) as increased heterogeneity has been shown to correlate with poor patient outcomes (Mroz et al. 2013; Mahrooghy et al. 2015). Typically the only time during the course of disease when tumor tissue is assessed by molecular methods is at diagnosis, and sometimes at progression. Access to solid tumor specimens at multiple time points over the course of a patient's therapy is often impossible and limits the clinical applicability of such testing for real‐time monitoring of a patient's disease.

Circulating tumor cells (CTCs) are known to shed into peripheral blood by many solid tumors (Pantel and Speicher 2015), and therefore provide an additional and less invasively accessible source of tumor material that can be collected in a serial fashion. CTC presence and persistence, as determined by the CellSearch System (Janssen Diagnostics, Raritan, NJ), the only FDA‐approved CTC enumeration and enrichment platform, have been associated with decreased progression‐free and overall survival in patients with metastatic breast, colorectal, and prostate cancer (Cristofanilli et al. 2004; de Bono et al. 2008; Cohen et al. 2008). Yet, thus far, most studies have focused on CTC enumeration rather than genetic characterization.

Several issues have limited the clinical application of next‐generation sequencing (NGS) to CTCs, including low CTC numbers and thus inadequate amounts of genetic starting material, as well as low purity of current CTC enrichment approaches. CTCs recovered from a tube of patient blood are quite rare relative to the prevalence of tumor cells in a resected solid tumor surgical specimen. Even in the metastatic setting, the number of CTCs detected by CellSearch is typically in the tens or hundreds for a 7.5 mL tube of whole blood (Allard et al. 2004). Given that a single cell contains 6–7 pg of DNA, pooling of the genomic DNA from even hundreds of CTCs would yield an amount of DNA well below the threshold input requirement for existing commercially available NGS platforms. To address this, whole genome amplification (WGA) can be used to generate sufficient amounts of starting material for sequencing, even when performed on few or single cells (Zong et al. 2012; Heitzer et al. 2013; Carpenter et al. 2014; Yu et al. 2014; Kelley et al. 2015). However, the white blood cell background resulting from currently available enrichment platforms is typically in the range from 10^3^ to 10^4^ (Sieuwerts et al. 2009), necessitating further enrichment or purification to achieve sufficient purity for clinical NGS platforms. We and others have used dielectrophoretic capture on the DEPArray (Silicon Biosystems, San Diego, CA) to purify and pool enriched CTCs (Carpenter et al. 2014; Polzer et al. 2014). However, this approach further prolongs processing time, often beyond the 24‐h window recommended for processing of unpreserved blood. Many fixatives, which can preserve blood for 72 h or more, can damage DNA by inducing nucleic acid cross‐linking (Srinivasan et al. 2002) and can be incompatible with commercially available WGA approaches, such as Qiagen REPLI‐g (Qiagen, Valencia, CA), or can increase WGA error rate (Carpenter et al. 2014). This incompatibility of patient sample preservation with WGA has served to further limit the application of NGS to CTCs.

In this study, we describe a novel approach for the isolation and NGS of rare tumor cells. Using breast cancer cell line spiking experiments, we demonstrate the feasibility of using dielectrophoretic capture of preserved CellSearch‐enriched CTCs, and combine this isolation approach with WGA as well as NGS using the Illumina TruSeq Amplicon Cancer Panel (TSACP; Illumina, San Diego, CA), a multiplexed targeted resequencing approach to NGS of cancer‐related genes. This study establishes the feasibility of a workflow for clinically relevant monitoring of tumor genetics in real time and over the course of a patient's therapy.

## Materials and Methods

### Cell isolation and purification

**Figure 1:**
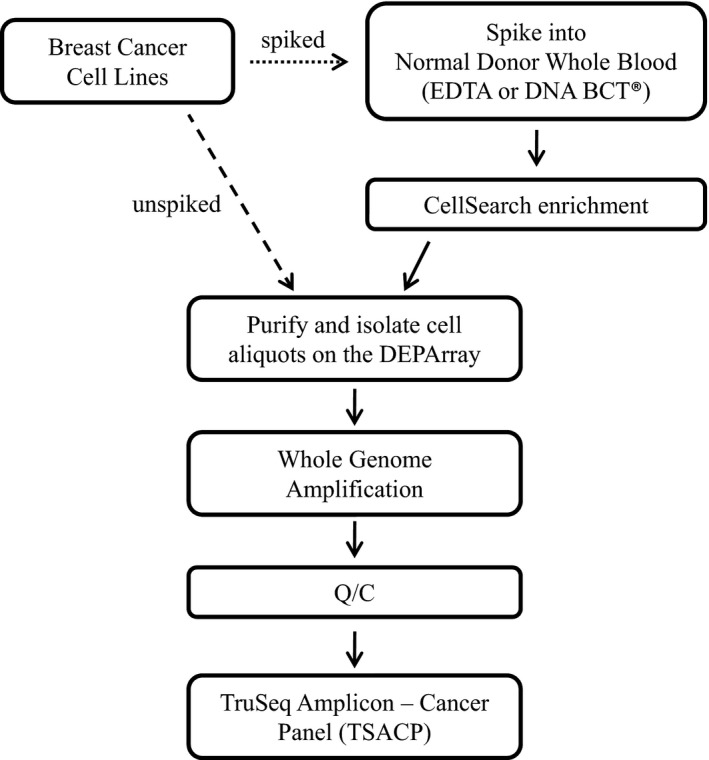
Workflows used to develop an approach for enrichment, purification, and sequencing of rare tumor cells. Here, we used unspiked and spiked cells to assess the feasibility of rare cell isolation combined with next‐generation sequencing. Initial experiments utilized the DEPArray to directly generate pools of unspiked cells (workflow depicted by dashed arrow). Subsequent experiments utilized spiked normal donor blood (depicted by right‐facing dotted arrow), in order to more closely mimic a process for circulating tumor cells isolation from patient blood.

Human breast cancer cell lines HCC1954 (Basal A subtype), MCF7 (luminal subtype), and MDA‐MB‐453 (luminal subtype) were purchased from ATCC (http://www.atcc.org), and normal donor whole blood was obtained from healthy volunteers at the University of Pennsylvania after obtaining written informed consent, according to the Declaration of Helsinki. The cell lines were maintained in the following conditions: the HCC1954 cell line in 5% CO_2_ incubation at 37°C in complete RPMI‐1640 (Mediatech, Manassas, VA), the MCF7 cell line in 5% CO_2_ at 37°C in complete EMEM (American Type Culture Collection, Manassas, VA), and the MDA‐MB‐453 cell line in 0% CO_2_ at 37°C in complete Leibovitz's L‐15 (Life Technologies, Carlsbad, CA). All cell lines were routinely mycoplasma tested. Adherent cell lines were grown to 75–80% confluence, trypsin‐treated for dissociation from the flask surface, and either processed unspiked on the DEPArray or 10,000–100,000 cells were spiked in 7.5 mL normal donor whole blood in a BD Vacutainer^®^ EDTA (Becton, Dickinson and Company, Franklin Lakes, NJ) or Streck Cell‐Free DNA BCT^®^ (Streck, Omaha, NE) tube (Fig. 1). EDTA tubes were processed within 24 h, and DNA BCT^®^ tubes within 72 h of blood draw. The DEPArray cartridge has a capacity of 40,000 cells, therefore requiring pre‐enrichment of the sample on the CellSearch prior to staining and processing on the DEPArray. To address this, the CellSearch Profile Kit (Janssen Diagnostics) was used for enrichment of cells of epithelial origin in whole blood. The post‐CellSearch samples were stained with NucBlue Live ReadyProbes Reagent (Life Technologies), monoclonal antibodies HER2‐PE (BioLegend, San Diego, CA) and EpCAM‐AlexaFluor647 (Cell Signaling, Danvers, MA) to detect cells of interest (HER2+/EpCAM+/CD45‐negative), and CD45‐AlexaFluor488 (Life Technologies) to distinguish white blood cells. Cells were centrifuged and resuspended in the appropriate cell culture medium as per the cell line. All staining and washing steps were performed in Protein LoBind Tubes (Eppendorf, Hauppauge, NY) to minimize cell loss. Next, 830 *μ*L of cell culture medium and 13 *μ*L of sample were loaded into the DEPArray cartridge. After the cartridge was placed in the DEPArray apparatus, nonuniform electrical fields were applied to isolate single cells. Prior to recovery, all cells were visually inspected in the Cell Browser (representative images in Fig. S1). Pools of 10, 20, 50, and 100 cells were captured into 0.2 mL microtubes (Applied Biosystems, Carlsbad, CA) followed by a PBS wash and supernatant removal, leaving the cells in approximately 5 *μ*L of volume. We also generated thousand‐cell pools for some experiments but these were not obtained on the DEPArray; rather, serial dilutions of the cell line were conducted. All cell pools were flash frozen on dry ice and stored at −20°C pending downstream analysis.

### Whole genome amplification and quality control

We conducted WGA using the REPLI‐g Single Cell Kit (Qiagen) according to the manufacturer's protocol. Controls for all experiments included unamplified genomic DNA as a positive control and a no‐cell tube containing only cell culture medium collected by DEPArray as a negative control. No false positive results were obtained for any of the 13 negative controls processed. All processing of DEPArray‐recovered cells was performed in two separate, dedicated clean rooms in order to prevent carryover of amplified DNA. Picogreen (Life Technologies) direct fluorescent staining was used as previously described (Carpenter et al. 2014) to quantify the concentration of DNA in the WGA product of captured cells. As an additional quality control (Q/C) measure, the Ampli1 QC Kit (Silicon Biosystems) was used to conduct multiplex PCR of 4 genes (chromosome 12p, 91 bp; chromosome 5q, 108–166 bp; chromosome 17p, 299 bp; chromosome 6q, 614 bp) as previously described (Carpenter et al. 2014). The workflow is illustrated in Figure 1.

### Statistics

When comparing Q/C measures for unspiked cells, or cells spiked into EDTA or DNA BCT^®^ tubes, we used analysis of variance (ANOVA), with pairwise comparisons using Tukey's studentized range test to adjust for multiple comparisons (Tukey 1949). To assess the association between the number of bands and other Q/C measures, including amount of WGA product and percent of fragments 100–600 bp, we used a Wilcoxon test.

### TruSeq Amplicon ‐ Cancer Panel: library preparation and sequencing

The Illumina TruSeq Amplicon – Cancer Panel (TSACP, FC‐130‐1008; Illumina) was utilized to capture mutational hotspots and surrounding exonic regions for 47 genes of interest. Oligonucleotide probes were designed such that each probe consists of complementary sequences to the nucleotides flanking the intended capture region and a tail of sequences that enables incorporation of sequencing adaptors. These oligonucleotide probes were manufactured and pooled together in a single tube. Next, we used the Agilent Genomic DNA TapeStation (Agilent Technologies, Santa Clara, CA) to assess DNA quality, according to the manufacturer's protocol, before loading into a 2200 TapeStation (Agilent Technologies).

The sequencing library preparations were performed following the Illumina guide and always including a no template sample as a negative control. Libraries were sequenced on an Illumina MiSeq instrument using 2 × 185 base pair reads. Sequencing data was analyzed by an internal clinically validated bioinformatics pipeline, which was created using both open source tools and custom algorithms (Daber et al. 2013). This pipeline was designed to extract essential performance statistics and to identify unique recurring mutations that eluded open source tools alone. *HER2* amplification was assessed by calculating mean *HER2* depth of coverage (DOC) relative to mean sample DOC. During assay validation, poor performing regions were removed from bioinformatics analysis, which resulted in 204 amplicons in 47 genes with a total size of ~35 kb defining the regions ultimately analyzed in this study. All variants listed are with reference to the hg19 Genome build.

### Sanger sequencing

PCR was performed as previously described (Carpenter et al. 2014) using the Ampli1 PIK3CA Sequencing Kit (Silicon Biosystems), and Sanger Sequencing was performed at the Children's Hospital of Philadelphia Nucleic Acid/Protein Research Core Facility (NAPCore) to detect the confirmed *PIK3CA* somatic mutations as listed in the COSMIC Cell Lines Project (http://cancer.sanger.ac.uk/cell_lines) for the MCF7 (c.1633G>A; E545K), HCC1954 (c. 3140A>G; H1047R), and MDA‐MB‐453 cell lines (c. 3140A>G; H1047R). All analyses were conducted using Sequencher (Gene Codes Corporation, Ann Arbor, MI).

## Results

### Next‐generation sequencing of amplified rare cell DNA from unspiked samples

We selected the Illumina TSACP approach for NGS of rare cells because of its coverage of genes of clinical relevance to breast and other cancers, and because it is often used, including by our own institution, to conduct clinical genomic analysis of solid tumor samples (Birner et al. 2014; Wilson et al. 2014; Azzato et al. 2015; Wong et al. 2015). This workflow would, therefore, provide a common platform for the analysis of matched circulating and resected tumor. One obstacle to the direct application of TSACP to liquid biopsies is that the genomic DNA contained in low numbers of CTCs is well below the 100 ng recommended input for TSACP. To overcome that problem, we employed REPLI‐g multiple displacement amplification WGA, an approach shown to be compatible with NGS (Farias‐Hesson et al. 2010; Hou et al. 2012). To assess the compatibility of this WGA approach with TSACP sequencing, we used cell lines representing two different breast cancer subtypes: the Basal A subtype HCC1954 cell line and the luminal subtype MCF7 cell line. Given that the majority of metastatic breast cancer patients with detectable CTCs will have 10 or more CTCs per 7.5 mL tube of whole blood (Allard et al. 2004; Cristofanilli et al. 2004; Paoletti et al. 2015), we took advantage of the precision with which dielectrophoretic capture can be conducted, and used the DEPArray to generate 10‐ and 100‐cell pools. Thousand‐cell pools were generated by serial dilution. In total, 22 pools of MCF7 and 64 pools of HCC1954 cells were generated (workflow in Fig. 1). DNA was extracted from these pools of cells and subjected to WGA.

**Figure 2:**
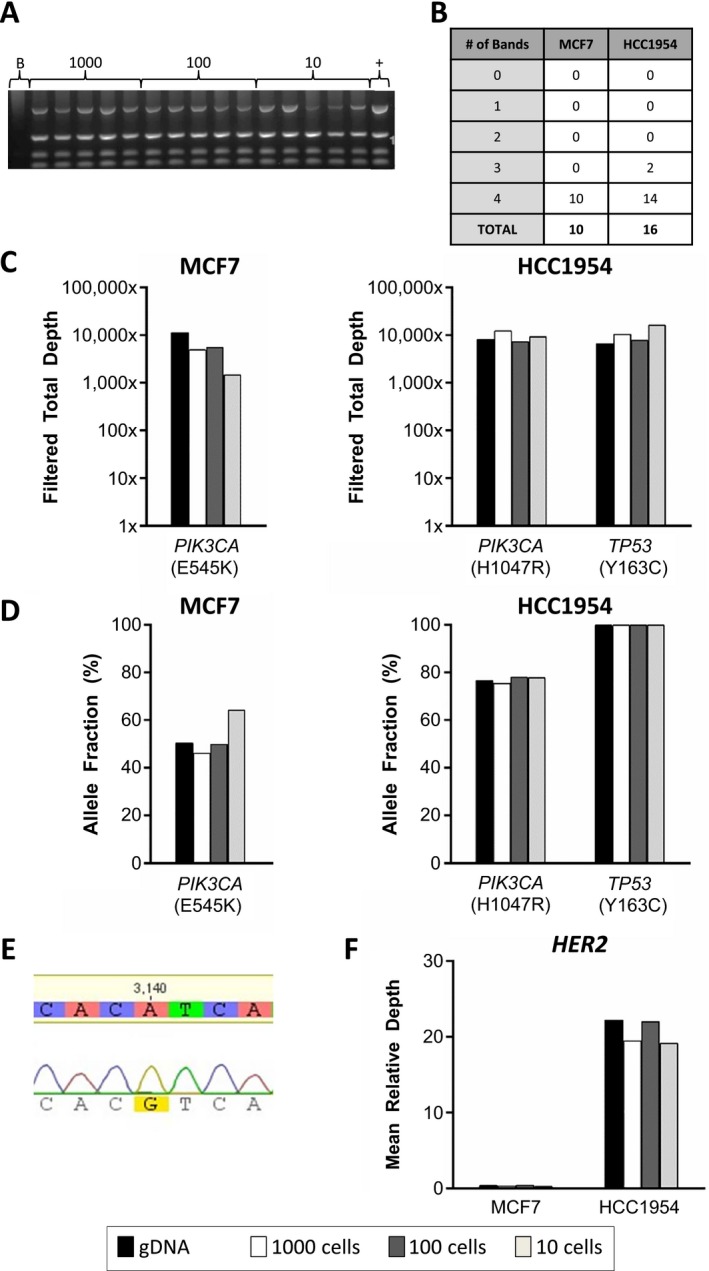
Next‐generation sequencing (NGS) of whole genome amplified DNA from unspiked cells. Ten pools of unspiked MCF7 and 16 pools of unspiked HCC1954 cells were generated. The 100‐ and 10‐cell pools were generated by DEPArray, while the 1000‐cell pools were generated using serial dilutions. DNA was extracted and whole genome amplification (WGA) performed. (A) An aliquot of the WGA product was assessed by multiplex PCR for the presence of four reference genes, as shown on the representative gel for 15 HCC1954 samples of the indicated pool sizes (1000‐, 100‐, or 10‐cell pools), a no‐cell “blank” (B), and positive control genomic HCC1954 DNA (+). (B) Shown are the number of bands detected by multiplex PCR for each sample. (C) Sequencing libraries were prepared for 3 MCF7 and 3 HCC1954 WGA products, as well as 1 genomic DNA sample for each cell line, for 8 samples in total. Shown here is the filtered total depth of coverage (DOC) at the DNA position of the variants in *PIK3CA* and *TP53* for each sample as detected by the TSACP platform. (D) Allele fraction for the indicated variants. (E) An additional aliquot of WGA product was submitted for Sanger sequencing to confirm the presence of the known heterozygous *PIK3CA* mutation. Shown is a representative chromatogram. *PIK3CA* mutations detected by TSACP NGS were confirmed by Sanger sequencing for all 8 samples. (F) *HER2* mean DOC relative to mean sample DOC.

In our initial experiments, we utilized unspiked cell lines in order to assess DNA and sequencing quality in minimally processed cells. Due to the high cost of NGS, a Q/C measure is critical when selecting for samples likely to be successful on the sequencer. We and others (Hou et al. 2012; Ni et al. 2013; Carpenter et al. 2014; Polzer et al. 2014) have shown that multiplex PCR can be used to predict sequencing success for Ampli‐1, Repli‐G, and other WGA approaches, thus avoiding the cost of NGS of low quality samples. To do this, an aliquot of each WGA product was assessed by multiplex PCR for the presence of four reference genes (representative image in Fig. 2A), with detection of two or more bands having been shown to predict higher sequencing success. We generated 26 unspiked cell line pools, including 10 MCF7 and 16 HCC1954, conducted Q/C, and detected four bands for 24 of 26 total samples (Fig. 2B). After Q/C, we selected the WGA product from six cell pools, as well as one genomic DNA (gDNA) sample for each of the two cell lines, for eight samples all together for NGS. Libraries were prepared and sequenced on the Illumina MiSeq. As shown in Figure 2C, all regions met our 250X minimum threshold for filtered total DOC, across the eight samples for the known disease‐associated variants in *PIK3CA* and *TP53*. Variants were detected at the allele fraction shown in Figure 2D. To further confirm the presence of the respective *PIK3CA* point mutations, we performed Sanger sequencing and detected the relevant mutation in all eight samples (representative chromatogram in Fig. 2E). Finally, we evaluated the increase in copy number for *HER2*, a gene amplified in 25–30% of breast cancers, and associated with aggressive disease and poor prognosis (Slamon et al. 2001; Vogel et al. 2002). As shown in Figure 2F, the four samples from the *HER2*‐unamplified MCF7 cell line had a mean relative depth within normal ranges (normal copy number = 1X). In contrast, a mean relative depth of 20.72X (SD = 1.41, range 19.15X–22.22X) was observed for *HER2* in the HCC1954 samples, reflecting the known amplification status. Taken together, these results demonstrate that our approach for WGA of DNA extracted from rare unspiked cells is compatible with TSACP NGS, and that known missense mutations and amplification events can be detected with our approach.

### Assessment of tumor cell DNA quality for unspiked and spiked samples

We next applied our approach for rare cell WGA and NGS to tumor cells isolated from spiked whole blood. For spiked blood, we used two different blood collection tubes, EDTA and DNA BCT^®^ tubes. To date, most reports of NGS of cells isolated from patient blood have used EDTA tubes for sample collection (Hou et al. 2012; Xu et al. 2012; Lohr et al. 2014; Zhang et al. 2014). However, to minimize cell degradation, EDTA tubes need to be processed within 24 h of blood draw, thus limiting opportunities for batching clinical patient samples. While the fixative paraformaldehyde is commonly used, we and others have shown this cell preservation method to lead to higher rates of sequencing errors (Swennenhuis et al. 2013; Carpenter et al. 2014), and this fixative is not compatible with the REPLI‐g WGA approach used here. Therefore, for our cell line spiking experiments into whole blood, we selected the preservative‐containing DNA BCT^®^ tube for comparison with the commonly used EDTA tube. DNA BCT^®^ tubes contain a proprietary preservative known to stabilize not only genomic DNA but also the membrane of the cell, thus enhancing cell stability and viability for up to 7 days (Norton et al. 2013; Wong et al. 2013). EDTA tubes, commonly used in clinical phlebotomy labs, contain no preservative but do contain EDTA to inhibit red blood cell coagulation. In our experiments, blood collected into DNA BCT^®^ tubes were stored at room temperature for up to 72 h before processing, whereas spiked EDTA tubes were never stored for more than 24 h.

**Figure 3:**
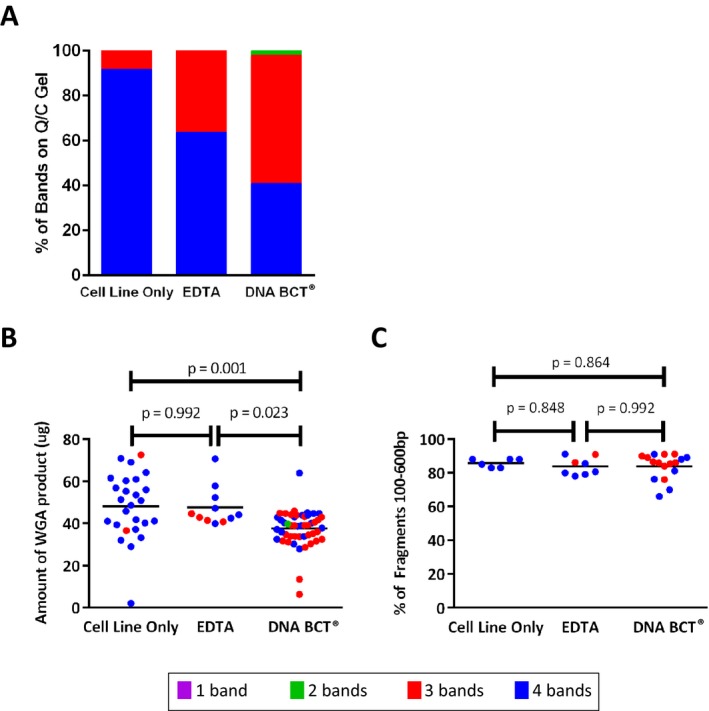
Comparison of whole genome amplification (WGA) product and library prep quality for 3 cell preparation methods. In these experiments, we used MCF7 or HCC1954 cells and the DEPArray to directly generate cell pools of unspiked cells (*n* = 26), or we spiked tumor cells into normal donor blood. For spiked samples, blood was collected into either EDTA (*n* = 11) or preservative‐containing DNA BCT ^®^ (*n* = 49) tubes (86 samples in total, including unspiked). Pools of 10, 20, 50, 100, and 1000 cells were generated. (A) Multiplex PCR of 4 genes was conducted and the indicated numbers of bands detected for each cell preparation method are displayed. (B) Picogreen was used to measure the amount of WGA product for an aliquot of each sample (*n* = 86), thus ensuring sufficient input material for library preparation and sequencing. (C) Libraries were prepared for 31 of the 86 samples, and the Tapestation‐based percentage of post‐library prep fragments that fall within 100–600 bp was measured. Color of dot for panels B and C indicates number of bands detected by Q/C as depicted in panel A.

Prior to sequencing, we wanted to quantitatively assess whether there were differences in the amount and quality of DNA when comparing unspiked cells, such as those used in the experiments described above, or spiked cells into either EDTA or DNA BCT^®^ tubes. As mentioned previously, pre‐NGS Q/C measures are essential for reducing the chance of conducting sequencing of low quality samples. To assess and compare the quality of DNA, unspiked HCC1954 and MCF7 cells were directly collected by DEPArray (*n* = 26; cell line only) as described above, or spiked into normal donor whole blood contained in either DNA BCT^®^ (*n* = 49) or EDTA (*n* = 11) tubes. For spiked samples, we used a two‐step approach for CTC capture (see Fig. 1 for workflow diagram). After isolating CTCs by CellSearch, the enriched fraction was immediately removed from the CellSearch AutoPrep tube, stained, and injected into the DEPArray cartridge for further processing. Pools of 10, 20, 50, 100, or 1000 cells were isolated, and WGA performed. Just as was described above for unspiked samples, 4‐gene multiplex PCR (representative image in Fig. 2A) was conducted for all 86 samples, and in these experiments comparing cell preparation methods, all of the 86 WGA products tested had two or more bands detected on the Q/C gel, and 85 of 86 had either three or four bands (Fig. 3A). Given the possibility that the two‐step CellSearch/DEPArray cell isolation process might affect cell quality, and that preservatives have been shown to affect DNA integrity, we added two additional pre‐NGS Q/C measures to our workflow. We first wanted to ensure that each WGA reaction had generated not only a sufficient amount of product for library preparation (TSACP recommended minimum input = 100 ng) but also excess product which could be used in the event of a failure in library preparation or sequencing, or for other forms of sequencing to validate the presence of detected variants. To do this, we used Picogreen to quantitate the total amount of DNA and found that all 86 samples contained 2 *μ*g or more DNA, and that 83 out of 86 samples contained 20 *μ*g or more DNA (Fig. 3B). While there was a significantly lower amount of DNA from DNA BCT^®^ samples (37.6 ± 8.4 *μ*g) than from EDTA (47.6 ± 8.9 *μ*g, *P* = 0.023) or cell line only (48.1 ± 5.2 *μ*g, *P* = 0.001), all samples were well in excess of the minimum DNA required for library preparation, and there was no significant difference comparing cell line only with EDTA (*P* = 0.992). Using a Wilcoxon test, we found no relationship between the number of bands detected by multiplex PCR (as indicated in Fig. 3B by the color of the dots) and the amount of WGA product for cell line only (*P* = 0.70), EDTA (*P* = 0.19), and DNA BCT^®^ (*P* = 0.14).

Once DNA concentration had been verified, 31 samples were selected for library preparation. To quantitatively assess the integrity of each library, and as an additional Q/C step, we analyzed the size of DNA fragments for each sample by measuring the percentage of fragments with a length of 100–600 bp. As shown in Figure 3C, and using ANOVA with Tukey's test as above, there was no significant difference between DNA BCT^®^ samples (84.27 ± 7.35%) and EDTA (83.94 ± 4.91%, *P* = 0.992), DNA BCT^®^ and cell line only (85.83 ± 2.27%, *P* = 0.864), or EDTA and cell line only (*P* = 0.848). Once again, samples with either three or four bands detected by multiplex PCR were tested, and using a Wilcoxon test, we found no relationship between the number of bands detected by multiplex PCR and fragment size for EDTA (*P* = 0.18) or DNA BCT^®^ (*P* = 0.22). No statistic was calculated for cell line only as all samples had four bands by Q/C gel. Of the 31 samples submitted for sequencing, all but two sequenced successfully. One failed sample had four Q/C bands, and the other had three Q/C bands as detected by multiplex PCR. Taken together, the quality control measures described above suggest that extended sample preservation using the DNA BCT^®^ tube results in the same DNA quality as that isolated from EDTA tubes, and is compatible with WGA and the preparation of high‐quality libraries. This demonstrates that DNA BCT^®^ tubes can be used to accommodate workflow requirements for a clinical NGS laboratory.

### Compatibility of blood sample preservation and NGS

**Figure 4:**
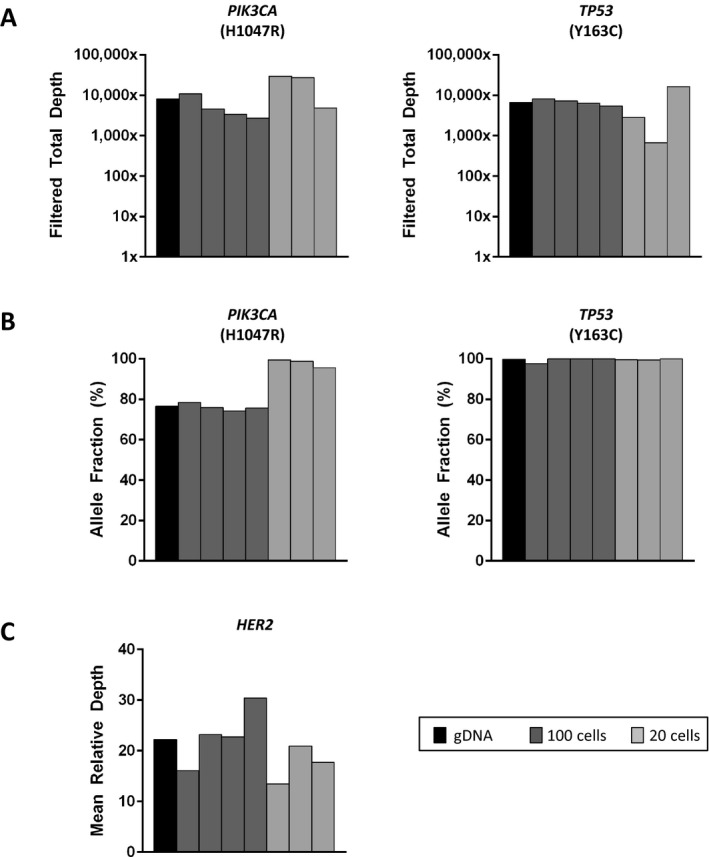
Next‐generation sequencing (NGS) of rare cells isolated from unpreserved spiked whole blood. We spiked HCC1954 cells into EDTA tubes containing normal donor blood, and processed within 24 h. We conducted CellSearch‐based enrichment, and DEPArray‐based isolation of tumor cell pools, and combined this with the whole genome amplification and NGS steps described above. We prepared libraries and sequenced seven pools of HCC1954 cells. Cell pool sizes of 100 (dark gray) and 20 (light gray) were used, with gDNA shown in black. (A) Shown are the filtered total depth of coverage (DOC) achieved for *PIK3CA* (left panel) and *TP53* (right panel), (B) allele fractions, and (C) *HER2* mean DOC relative to mean sample DOC.

**Figure 5:**
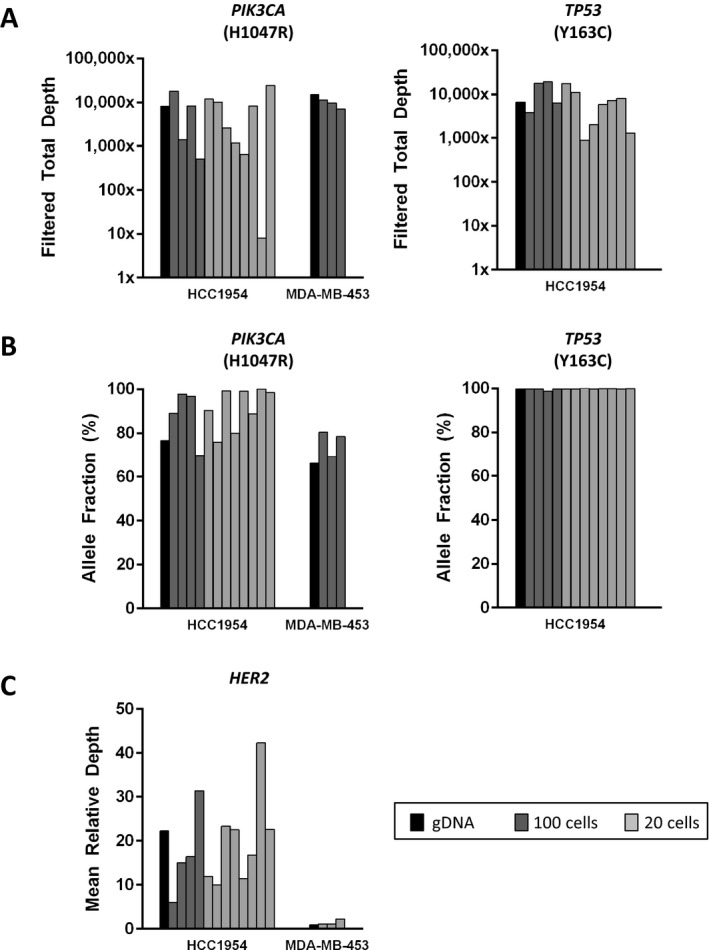
Next‐generation sequencing (NGS) of rare cells isolated from preserved spiked whole blood. Here, we spiked HCC1954 or MDA‐MB‐453 cells into normal donor blood preserved in DNA BCT ^®^ tubes for up to 72 h. As described above, we conducted CellSearch‐ and DEPArray‐based isolation of rare cells, whole genome amplification, and NGS. We prepared libraries and sequenced 15 total samples, including pools of HCC1954 (*n* = 12) and MDA‐MB‐453 (*n* = 3) cells. Cell pool sizes of 100 (dark gray) and 20 (light gray) were used, with gDNA shown in black. (A) Shown are the filtered total DOC achieved for *PIK3CA* (left panel) and *TP53* (right panel), (B) allele fractions, and (C) *HER2* mean depth of coverage (DOC) relative to mean sample DOC.

Having confirmed the quality and quantity of whole genome amplified rare cell DNA from spiked whole blood, we next performed sequencing to determine whether differences in preservation methods affect NGS. To do this, we sequenced 22 libraries prepared from the WGA product of spiked blood, including seven from EDTA and 15 from DNA BCT^®^ tubes, and compared to results for genomic DNA. For the seven EDTA samples, pools of 20 or 100 HCC1954 cells were evaluated, with filtered total DOC shown in Figure 4A and allele fraction in Figure 4B for *PIK3CA* and *TP53* variants. All samples achieved a minimum filtered total DOC of 250X, and the multiplex PCR, Picogreen, and TapeStation quality control results are included among those shown in Figure 3. *HER2* copy number was evaluated for this *HER2*‐amplified cell line, and relative *HER2* sequencing depths were found to range from 13.44 to 30.38 (Fig. 4C). Next, we applied WGA and NGS to cells captured from spiked DNA BCT^®^ tubes. In addition to HCC1954 (8 20‐cell and 4 100‐cell pools; harbors missense mutations in PIK3CA (c. 3140A>G; H1047R) and TP53 (c. 488A>G; Y163C)), we applied our approach to a third cell line, MDA‐MB‐453 (3 20‐cell pools), which harbors a PIK3CA missense mutation (c. 3140A>G; H1047R) but is TP53 wild‐type (Hollestelle et al. 2007). All Q/C results for the MDA‐MB‐453 samples are shown in Table S1. As shown in Figure 5A, 14 out of 15 DNA BCT^®^ samples achieved sufficient filtered total DOC for *PIK3CA* (left panel) and *TP53* (right panel) variants. Despite having passed our WGA and library preparation Q/C measures, one 20‐cell HCC1954 sample was below the 250X threshold, with a filtered total DOC of 8X. Finally, we evaluated *HER2* copy number in each of the 15 samples, which confirmed the *HER2*‐amplification of the HCC1954 cell line and showed no amplification in the HER2‐negative MDA‐MB‐453 cell line (Neve et al. 2006; Vranic et al. 2011). As shown in Figure 5C, *HER2* amplification was detected for all 12 HCC1954 samples, with relative depth ranging from 5.99 to 42.32. As expected, for the 3 MDA‐MB‐453 samples, *HER2* relative depth was within normal ranges. These results confirm the compatibility of our WGA and blood preservation approaches with a clinically relevant NGS platform.

## Discussion

Here, we have presented proof of concept for a clinically relevant approach for NGS that is applicable to rare CTCs. While comprehensive genetic analysis of solid tumors is routinely conducted in our own and other clinical laboratories, clinical molecular analysis of liquid biopsies for solid tumors is not currently widespread. The use of blood tests for cancer patient molecular monitoring, a practice that would minimize or even obviate the need for repeat surgical biopsies, has been restricted by low numbers of CTCs, long sample processing timelines, and incompatibility of common sample fixatives with downstream molecular analysis. Yet, there is an urgent need for the development of such tests. Surgical access to tumor, especially in the metastatic setting, may be limited or impossible. Moreover, metastatic disease is known to be highly heterogeneous, and biopsies of some or all metastatic sites are impractical. Promising therapies targeting specific gene mutations in breast and other cancers have recently been reported (Valero et al. 2011; Saura et al. 2014; Shaw et al. 2014; Swain et al. 2015), and yet robust blood‐based tests for the detection of such biomarkers have not been established in the clinical setting. Here, we focus on a commercially available sequencing approach with broad genomic coverage and established clinical relevance (Birner et al. 2014; Wilson et al. 2014; Azzato et al. 2015; Wong et al. 2015). We combine NGS with WGA and sample preservation into a single pipeline for clinically relevant variant detection. Using this approach on spiked normal donor blood samples, we focused on three of the most prevalently mutated genes in breast cancer, *PIK3CA*,* TP53*, and *HER2* (Cancer Genome Atlas Network 2012), and were able to demonstrate the detection of known disease‐associated variants in these genes across three breast cancer cell lines and for 20‐ and 100‐cell pools. This suggests an approach that can be applied to the rare CTCs present in the blood of breast and other cancer patient samples.

In our experiments, we purified and molecularly characterized cell pools ranging from 10 to 1000 cells, as these numbers of cells are representative of what can be isolated from a single tube of blood by CellSearch for tumors of epithelial origin such as breast, lung, and prostate cancer (Allard et al. 2004). Liquid biopsies have begun to gain traction in the metastatic setting for enumeration of CTCs but will only realize their full potential when molecular analysis can be applied to the fewer than 20 cells likely to be detectable for patients with minimal residual disease. Use of serial blood‐draws for monitoring of patients on therapy, and also for variant detection in early stage disease, will require the ability to interrogate very few cells (Ignatiadis et al. 2011). The work presented here demonstrates the feasibility of our approach for small pools of cells and opens the door to applying the approach to fewer or even single cells. Adapting the above described approach for clinical application will also require larger scale testing on patient samples. For the 15 spiked samples to which we applied our workflow, the known *PIK3CA*,* TP53*, and *HER2* variants were reliably detected for all but one sample in which the filtered total DOC was below 250X for *PIK3CA*. One explanation for this is the propensity of the WGA process to have certain allelic biases in unique reactions of the same starting material (Pinard et al. 2006). While we were able to achieve a high level of accuracy, additional testing and optimization will be needed to accurately assess sensitivity and specificity.

In some cancers, including pancreatic and non‐small cell lung cancer (Allard et al. 2004), CTCs are very difficult, and often impossible, to detect by CellSearch. As the CellSearch technology evolves, and new approaches become available to improve sensitivity of CTC isolation for these cancers, as well as melanoma, glioblastoma, and other cancers not originating from epithelial tissue, it will be important to evaluate the compatibility of those CTC isolation approaches with the WGA and NGS steps we have described. Improvements in CTC isolation sensitivity will also need to be accompanied by improved throughput times, as the combination of CellSearch‐based and DEPArray‐based CTC enrichment described here takes several hours to complete. However, a new version of the DEPArray introduced since the above experiments were conducted already decreases processing time and increases sample capacity, thus improving clinical testing relevance.

Here, we have demonstrated the feasibility of a liquid biopsy for NGS, thus moving the field beyond simple enumeration approaches. While CellSearch‐based CTC enumeration has been firmly established as an independent prognostic measure for breast, prostate, and colorectal cancer (Cohen et al. 2008; Coumans et al. 2012; Pierga et al. 2012), a recent study of metastatic breast cancer patients reported that switching therapy based on CTC count alone did not improve overall patient survival (Smerage et al. 2014). In contrast, our approach offers breast and other cancer patients the hope of noninvasive tumor monitoring that may more comprehensively assess tumor genomics and improve the precision for predicting response to therapy and risk of relapse. Preservation of patient blood samples in a manner compatible with NGS and also with extended storage times means that blood samples can be shipped from satellite clinical locations to a central testing facility, and future studies will assess whether samples can be reliably sequenced after storage for longer than 72 h. The DNA BCT^®^ tube has been shown to be compatible with the extraction and NGS of cell‐free DNA (Norton et al. 2013; Wong et al. 2013), suggesting the eventual possibility of sequencing both cell‐free and cellular DNA from a single tube of blood. Moreover, this workflow can likely be adapted to apply to other types of cancer patient samples with rare tumor cells, including pleural effusions, ascites, and bone marrow aspirates. In summary, this workflow enables comprehensive molecular analysis of rare circulating cells thus providing real‐time monitoring of patient tumor using liquid biopsies.

## Conflict of Interest

None declared.

## Supporting information


**Figure S1.** DEPArray‐isolated single cells from DNA BCT^®^ tubes.

**Table S1.** Q/C results for three 20‐cell pools of MDA‐MB‐453.
