# Supplementary material for: A novel approach for next‐generation sequencing of circulating tumor cells
Source: Mol Genet Genomic Med. 2016 Feb 28;4(4):395–406. doi: 10.1002/mgg3.210 (PMC4947859; doi:10.1002/mgg3.210)
Supplement: Supplementary file 1 — Figure S1. DEPArray‐isolated single cells from DNA BCT® tubes. [file MGG3-4-395-s001.pptx]

## Slide 1
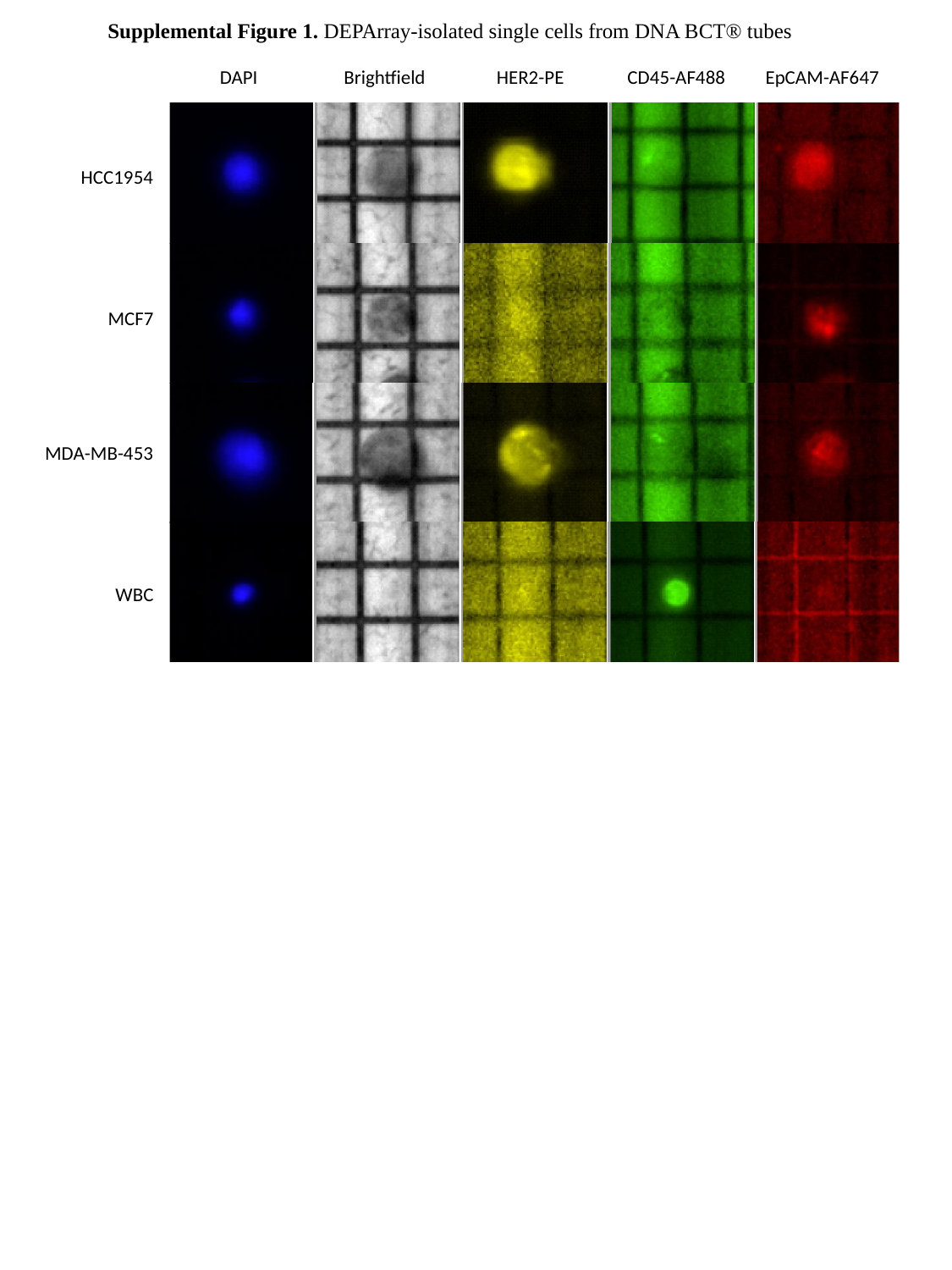

Supplemental Figure 1. DEPArray-isolated single cells from DNA BCT® tubes
| DAPI | Brightfield | HER2-PE | CD45-AF488 | EpCAM-AF647 |
| --- | --- | --- | --- | --- |
HCC1954
MCF7
MDA-MB-453
WBC
