# Supplementary material for: A novel approach for next‐generation sequencing of circulating tumor cells
Source: Mol Genet Genomic Med. 2016 Feb 28;4(4):395–406. doi: 10.1002/mgg3.210 (PMC4947859; doi:10.1002/mgg3.210)
Supplement: Supplementary file 2 — Table S1. Q/C results for three 20‐cell pools of MDA‐MB‐453. [file MGG3-4-395-s002.pptx]

## Slide 1
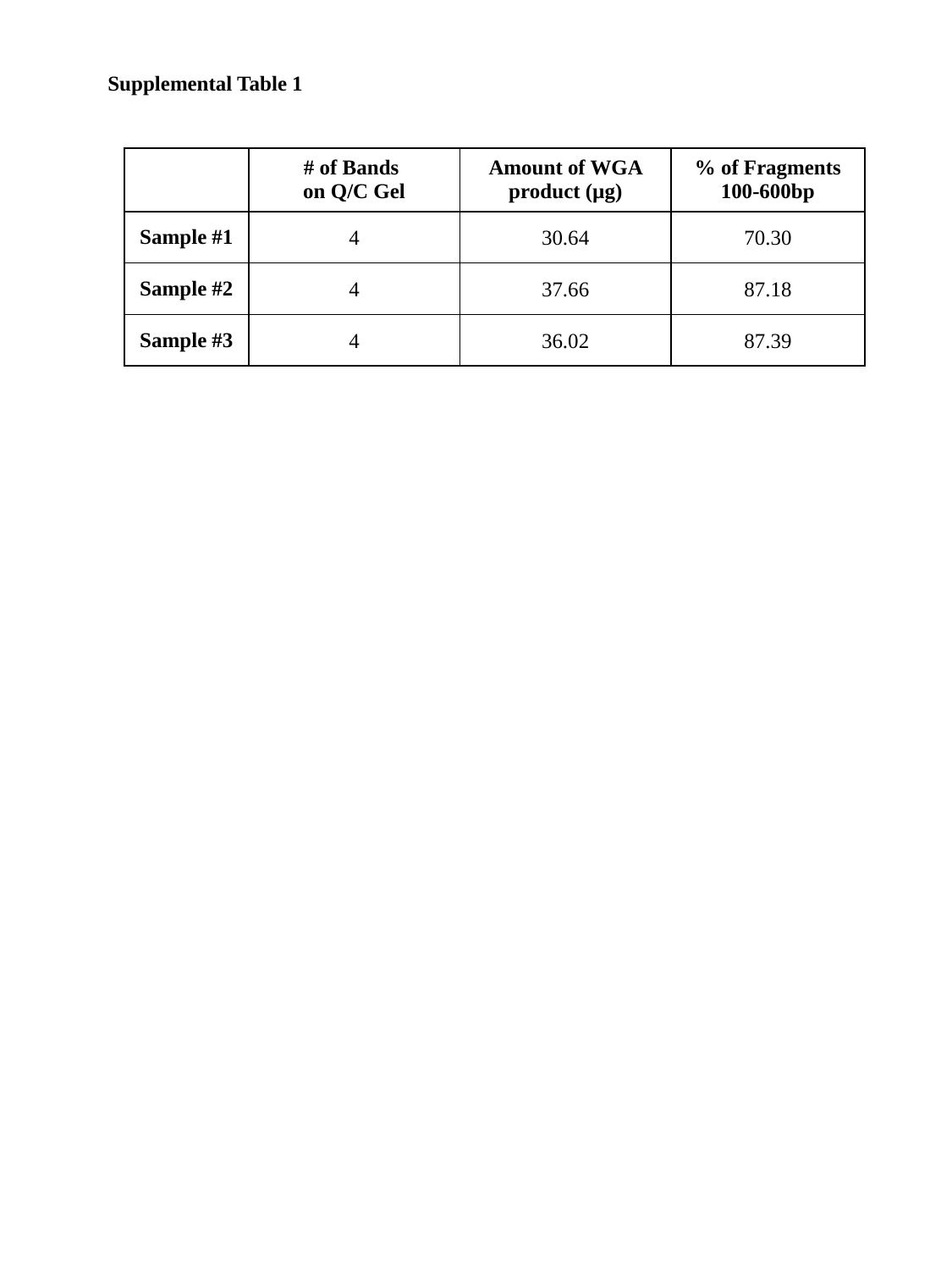

Supplemental Table 1
| | # of Bands on Q/C Gel | Amount of WGA product (µg) | % of Fragments 100-600bp |
| --- | --- | --- | --- |
| Sample #1 | 4 | 30.64 | 70.30 |
| Sample #2 | 4 | 37.66 | 87.18 |
| Sample #3 | 4 | 36.02 | 87.39 |
